# Genome-wide identification and functional analysis of long non-coding RNAs in *Chilo suppressalis* reveal their potential roles in chlorantraniliprole resistance

**DOI:** 10.3389/fphys.2022.1091232

**Published:** 2023-01-09

**Authors:** Shuijin Huang, Dong Jing, Lu Xu, Guanghua Luo, Yanyue Hu, Ting Wu, Yao Hu, Fei Li, Kang He, Wenjing Qin, Yang Sun, Hui Liu

**Affiliations:** ^1^ Institute of Plant Protection, Jiangxi Academy of Agricultural Sciences, Nanchang, China; ^2^ Institute of Insect Sciences/Ministry of Agriculture Key Laboratory of Molecular Biology of Crop Pathogens and Insect Pests, College of Agriculture and Biotechnology, Zhejiang University, Hangzhou, China; ^3^ Institute of Plant Protection, Jiangsu Academy of Agricultural Sciences, Jiangsu Key Laboratory for Food and Safety-State Key Laboratory Cultivation Base of Ministry of Science and Technology, Nanjing, China; ^4^ Institute of Animal Husbandry and Veterinary Medicine, Jiangxi Academy of Agricultural Sciences, Nanchang City, China; ^5^ Institute of Soil Fertilizer and Environmental Resource, Jiangxi Academy of Agricultural Sciences, Nanchang, China; ^6^ Institute of Red Soil and Germplasm Resources in Jiangxi, Nanchang, China

**Keywords:** chlorantraniliprole resistance, long non-coding RNAs, adjacent protein-coding genes, genome-wide identification, *Chilo suppressalis*

## Abstract

Long non-coding RNAs, referred to as lncRNAs, perform essential functions in some biological processes, including reproduction, metamorphosis, and other critical life functions. Yet, lncRNAs are poorly understood in pesticide resistance, and no reports to date have characterized which lncRNAs are associated with chlorantraniliprole resistance in *Chilo suppressalis*. Here, RNA-seq was performed on two strains of *C. suppressalis* exposed to chlorantraniliprole: one is a susceptible strain (S), and the other is a resistant strain (R). In total, 3,470 lncRNAs were identified from 40,573 merged transcripts in six libraries, including 1,879 lincRNAs, 245 intronic lncRNAs, 853 sense lncRNAs, and 493 antisense lncRNAs. Moreover, differential expression analysis revealed 297 and 335 lncRNAs upregulated in S and R strains, respectively. Differentially expressed (DE) lncRNAs are usually assumed to be involved in the chlorantraniliprole resistance in *C. suppressalis*. As potential targets, adjacent protein-coding genes (within <1000 kb range upstream or downstream of DE lncRNAs), especially detoxification enzyme genes (cytochrome P450s, carboxyl/cholinesterases/esterases, and ATP-binding cassette transporter), were analyzed. Furthermore, the strand-specific RT-PCR was conducted to confirm the transcript orientation of randomly selected 20 DE lincRNAs, and qRT-PCR was carried out to verify the expression status of 8 out of them. *MSTRG.25315.3*, *MSTRG.25315.6*, and *MSTRG.7482.1* were upregulated in the R strain. Lastly, RNA interference and bioassay analyses indicated overexpressed lincRNA *MSTRG.7482.1* was involved in chlorantraniliprole resistance. In conclusion, we represent, for the first time, the genome-wide identification of chlorantraniliprole-resistance-related lncRNAs in *C. suppressalis*. It elaborates the views underlying the mechanism conferring chlorantraniliprole resistance in lncRNAs.

## Introduction

The striped stem borer (termed SSB), *Chilo suppressalis* Walker (Lepidoptera: Pyralidae), is one of the most destructive insect pests of rice in China, causing significant yield losses. The SSB larvae feed within plant stems, and damage leads to death of sheaths and cores, resulting in white tassels in rice (Sheng et al., 2003; He et al., 2013). Management of this insect pest mainly relies on insecticide application. Monosultap, triazophos, abamectin, and chlorantraniliprole are currently the most widely used insecticides in reducing the population of SSB (Shuijin et al., 2017). Chlorantraniliprole is the most effective of these options; it is effective against SSB and stem borers and is utilized most heavily in areas known to consistently have high densities of the pest (Sun et al., 2021; Dai et al., 2022). Chlorantraniliprole is an anthranilic diamide insecticide that functions *via* activation of the insect ryanodine receptors located on the sarcoplasmic reticulum in muscle cells, causing sustained release of calcium levels within the cytosol which leads to muscle contraction, paralysis, and eventual death of the organism (Lahm et al., 2005; Ebbinghaus-Kintscher et al., 2006). However, SSB have developed a low-level and medium-level (Su et al., 2014) resistance to chlorantraniliprole in a short time, eventually developing high-level resistance (Yao et al., 2017), followed very soon by extremely-high-level resistance (Sun et al., 2018).

Understanding of resistance mechanism is helpful in formulating more reasonable resistance management strategies. Target resistance can be determined by the interaction pattern between chlorantraniliprole and RyR (Guo et al., 2014). In SSB, four RyR mutants (I4758M, G4910E, I4891F, and Y4667D) were reported, and they were recently functionally confirmed (Yao et al., 2017; Sun et al., 2018; Huang et al., 2020). The metabolic detoxification was also tested already in chlorantraniliprole. Over-expression of cytochrome P450 genes, *CYP321F3*, *CYP6CV5*, *CYP32412*, and *CYP9A68*, caused chlorantraniliprole insensitivity (Xu et al., 2019). Increased esterase activity possibly improved chlorantraniliprole resistance as well (Sun et al., 2018). A genome-wide study discovered dozens of ATP-binding cassettes (ABCs) and found *ABCA1*, *-D2*, and *-H2* are upregulated in all three resistant SSB strains (Peng et al., 2021). However, these previous studies mainly focus on the analyses of protein-coding genes, whereas non-coding RNA genes associated with chlorantraniliprole resistance in SSB have not been characterized.

LncRNAs are an important component of >200 nT non-coding RNA (Quinn and Chang, 2016) and were previously regarded as “transcriptional noise” (Struhl, 2007). More recently, evidence has shown that they perform essential regulatory functions in various biological processes or events, like imprinting genomic loci, X-chromosome silencing, shaping chromosome conformation, and carcinogenesis (Angrand et al., 2015; Chen et al., 2016; Diederichs et al., 2016; Quinn and Chang, 2016). Because the genome is difficult to obtain, these in-depth mechanism studies are limited to humans, mice, and the other model species (Li et al., 2019). For insects, the recent progress in sequencing technology, genome-wide investigation, and preliminary functional analysis was the main focus in lncRNA studies (Zhu et al., 2017; Chang et al., 2020; Yang et al., 2021; Wu et al., 2022). They identified 1309, 4516, 6171, and 11978 lncRNAs in *Plutella xylostella* (Zhu et al., 2017), *Tribolium castaneum* (Yang et al., 2021), *Bactrocera dorsalis* (Meng et al., 2021), and *Spodoptera litura* (Shi et al., 2022), respectively. In addition, the functions of insect lncRNAs could be involved in RNAi pathways, wing development, response to heat stress, plant–insect interactions, and pesticide resistance (Bernabo et al., 2020; Chen et al., 2020; Guan et al., 2020; Shang et al., 2021; Zhu et al., 2021).

Non-coding RNAs, such as lncRNAs, are hypothesized to play much more important roles in multiple biological processes, and uncovering their function in pesticide resistance will lead to novel insights and expanded avenues for integrated pest management (IPM). In this study, lncRNAs of SSB in susceptible (S) and resistant (R) strains were obtained *via* next-generation sequencing (NGS), and the differentially expressed (DE) lncRNAs in both S and R strains were investigated and located in the genome. Subsequently, adjacent protein-coding genes within 1000 kb apart from DE lncRNAs were mapped in the genome, including P450s, carboxyl/cholinesterases/esterases, and ABC genes. Also, the detoxification enzyme gene family was further analyzed to study their possible association with chlorantraniliprole resistance. Moreover, RT-PCR and qRT-PCR were conducted to validate the selected DE lncRNAs. Lastly, RNAi and chlorantraniliprole bioassay were performed to explore the lncRNA functions in chlorantraniliprole resistance. These findings report the first characterization of lncRNAs in SSB and elaborate the knowledge on the existing mechanism of development of chlorantraniliprole resistance in non-coding RNAs.

## Materials and methods

### Insect strains

A susceptible strain (S) of SSB was sourced from the Institute of Plant Protection, Chinese Academy of Agricultural Sciences (Haidian District, Beijing), provided by Dr. Lanzhi Han and reared on an artificial diet (Shuijin et al., 2017) under constant laboratory conditions (temperature: 27°C ± 1°C, relative humidity: 70%–80%, and photoperiod: 16:8) without insecticides for over 50 generations. The S strain was screened under chlorantraniliprole treatment for over 30 generations, and the resistance ratio (RR) of the R strain was 110.4 compared to the S strain, as previously reported (Sun et al., 2018). The SSB of both S and R strains were reared on an artificial diet with or without chlorantraniliprole treatment.

### RNA extraction, library preparation, and sequencing

Total RNA was extracted from three to four 4th instar SSB larvae using the TRIzol reagent kit (Life Technologies, United States), following the manufacturer’s instructions. RNA quality was first checked by 1% (w/v) agarose gel electrophoresis, followed by a NanoDrop spectrophotometer (RNA model, California, United States). Lastly, Qubit 2.0 accurately quantified the RNA concentration. We finished the library construction and RNA-seq (Novogene, China). The total RNA of six samples (three independent biological replicates for S and R strains, respectively) was purified and qualified first, followed by the removal of rRNA using the Ribo-Zero kit (Epicentre, United States), the random disturbance of RNA in fragmentation buffer (NEB, United States), and the cDNA synthesis with 6-bp random hexamers. The purified double-stranded cDNA was repaired and adapter-ligated. Then, the cDNA library was enriched by PCR after being purified. Sequencing was performed on the NovaSeq 6000 platform.

### lncRNA identification, gene structural features, and differential expression analysis

To isolate SSB lncRNAs from the sequencing dataset, a computational pipeline was constructed, which was referred to report with minor modifications (Liu et al., 2017). At the very beginning, Trimmomatic software was used to filter low-quality reads (Bolger et al., 2014). The raw reads from the six libraries were then mapped to the SSB genome *via* TopHat (Trapnell et al., 2009). In detail, the reads from each library were first mapped to the scaffolds, and the junction outputs from every RNA-seq dataset were combined together to generate a “pooled junction set,” which was further used to map all of the reads from different RNA-seq datasets to the scaffolds *via* TopHat. This step supplied a junction set for Cufflinks (Trapnell et al., 2012). Then, the six datasets were combined into a whole transcriptome by Cuffcompare, according to the annotated genomic information. The transcripts that are longer than 200 nT and have more than two exons were reserved, and 24,267 transcripts were obtained in this step. At this point, the candidate protein-coding genes were excluded after blasting to the NR database (e < 001). Next, transcripts >300 nT ORF were removed by getorf software (http://emboss.sourceforge.net/apps/cvs/emboss/apps/getorf.html). Then, the protein-coding transcripts were predicted by Coding Potential Assessment Tool (CPAT), and other transcripts were used as the template to search in the Pfam database with HMMER software (Finn et al., 2015). The transcripts that have no conserved domain/motif potential were reserved, while other non-coding RNAs except lncRNAs, the known tRNAs, snoRNAs, snRNAs, and rRNAs were all removed by the Infernal and BLASTN search (Zhao et al., 2016), generating the final lncRNA sets.

Aligning lncRNAs with the SSB genome (Ma et al., 2020), we analyzed the lncRNA exon–intron structure by Geneious (Kearse et al., 2012) and also checked its distribution among the scaffolds.

The transcript enrichment of the identified lncRNAs was examined by counting reads and normalized by Cuffdiff software, using the *t-*test to display the significance of DE. The lncRNA expression level was detected with FPKM (fragments per kilobase of transcript per million fragments mapped) as an indicator. Q-value is the FDR-adjusted *p*-value. A lncRNA which meets these criteria will be defined as specifically expressed: 1) the value of |log2 fold-change|≥1; 2) *p*-value <.01; and 3) q-value<.01. The fold change refers to the ratio of expression amount between two strains. The lncRNAs obtained by the differential expression analysis are displayed in the form of a volcano plot.

### Strand-specific RT-PCR

Sample preparation, RNA extraction, and quality control are as described previously. Three reactions during the cDNA synthesis are forward (F) primer with reverse transcriptase (RT), reverse (R) primer with RT, and F + R primers without RT. The primers for RT-PCR (Supplementary Table S1) were designed using http://www.idtdna.com/Scitools/and synthesized by Sangon Biotech Co., Ltd. (Shanghai, China).

### RNA isolation, cDNA synthesis, and quantitative real-time PCR

The extraction and purification of total RNA were described previously. cDNA was obtained by using the PrimeScript^®^RT Master Mix kit (TaKaRa, Japan) with 500 ng of total RNA as the template. qRT-PCR was carried out with a SYBR Premix Ex Taq kit (TaKaRa, Japan) on an ABI 7300 real-time PCR system (Thermo Fisher Scientific, United States) as described as follows: 30 s denaturation at 95°C, 40 cycles of 5 s at 95°C, and 31 s at 60°C, 15 s at 95°C, 15 s at 60°C, and 15 s at 95°C again. *Actin A1* was used as an internal reference. Data were analyzed by the 2^−ΔΔCt^ method (Pfaffl 2001). The primers used are shown in Supplementary Table S2.

### Expression profile across life-time and RNAi assay of the lincRNA *MSTRG.7482.1*

Samples of different development stages were collected including the egg, 1st, 2nd, 3rd, 4th, 5th, and 6th stages, pupae, and male and female moths. For each stage, 3–4 individuals were collected as one biological replicate, and four biological replicates were performed. These samples were then used to conduct RNA extraction, cDNA collection, and qRT-PCR for the lincRNA *MSTRG.7482.1*.

The chemically synthesized small interference RNA (siRNA) came from GenePharma Co., Ltd. (Shanghai, China) (see Supplementary Table S2 to find its sequence).The HPLC-purified double-stranded siRNAs were dissolved in diethylpyrocarbonate-treated water (Milli-Q-grade) to make a 4 mg/ml solution. Then, 1 μl (4 μg) of siRNA was injected into fourth-instar larvae with a micro-needle, pulling the needles from glass capillaries which have 1.0-mm outer diameter and 50-mm inner diameter by using a micropipette puller (Model P-87, Sutter Instruments Co., CA), keeping needles still for 30 s at the injection point to avoid siRNA leakage. The shuffled siRNAs were taken as negative control (Supplementary Table S2). About 20 to 30 specimens were examined for each treatment, with triplicate experiments. The fourth larvae were collected 24 and 48 h post-micro-injection to determine the efficiency of RNAi.

For RNAi assay, the larvae were removed onto an artificial diet without any pesticide treatment for 24 h post-injection. The larvae that died or whose movement was blocked due to mechanical injury were discarded. The remaining specimens were removed onto an artificial diet with chlorantraniliprole treatment, according to the LC_50_ dose in S or R strains for another 96 h, respectively. Lastly, these specimens were removed onto an artificial diet without treatment. The mortality was calculated day by day from the fourth day post-treatment with chlorantraniliprole.

### Data analysis

For the location of lncRNA and protein-coding genes on the genome of SSB, information on the chromosome length, lncRNA/gene density, and location of the lncRNA/gene to be located was collected. The R package RIdeogram was used to display the aforementioned information. Regarding the qRT-PCR result, Tukey’s test and one-way ANOVA statistical analysis were performed to compare the different gene expression levels between S and R strains.

## Results

### Identification and characterization of lncRNAs in SSB

**FIGURE 1:**
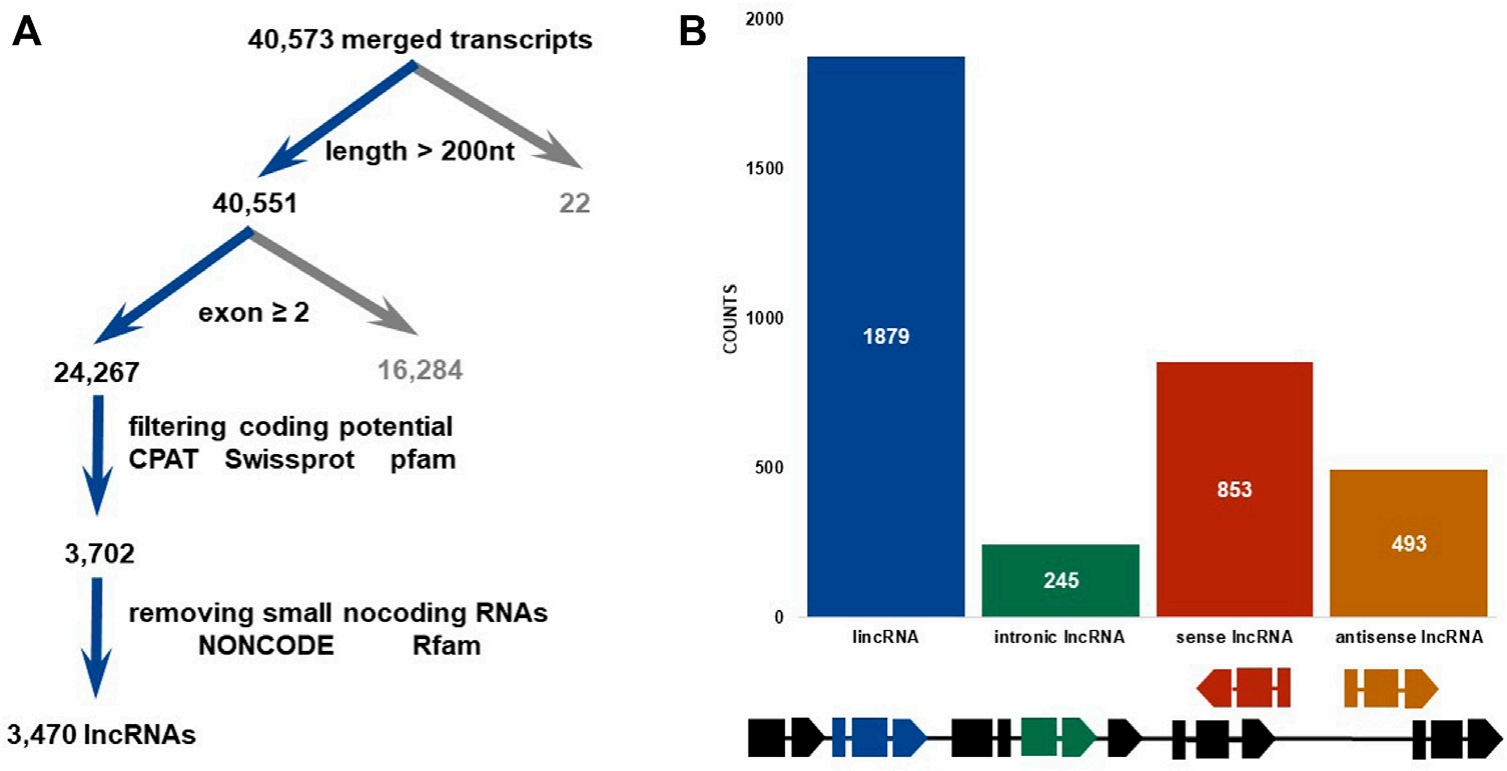
Identification and classification of lnc RNAs in SSB. **(A)** Brief computational pipeline for identifying lncRNAs. **(B)** Histogram of lncRNAs categorized as lincRNA (long intergenic non-coding RNA), intronic lncRNA, sense exon lncRNA, and antisense exon lncRNA. The schematic diagram at the bottom shows the differences of the gene structure of different types of lncRNAs in the genome.

**FIGURE 2:**
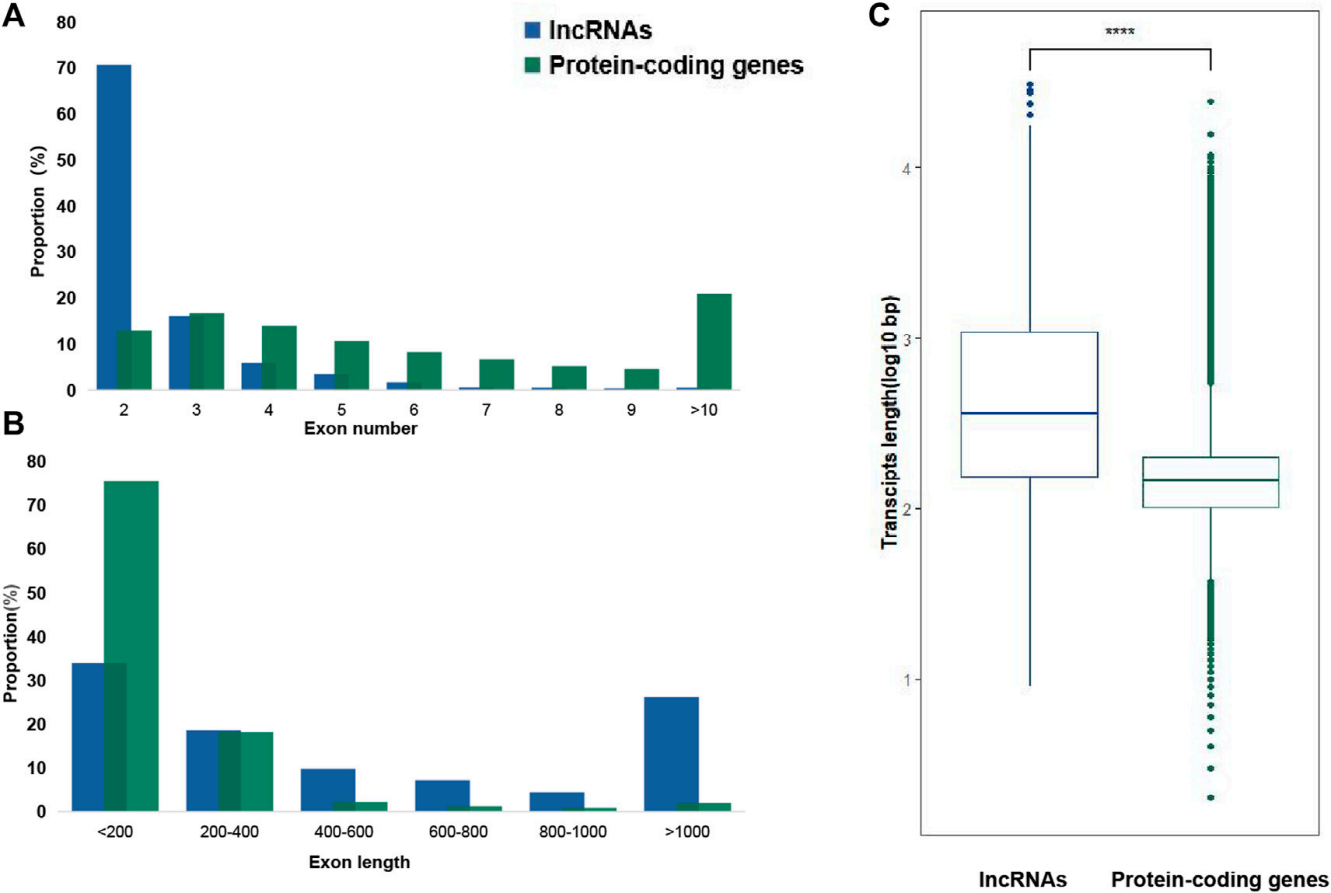
LncRNA gene structural features of SSB. **(A)** Exon numbers of lncRNAs and protein-coding genes. **(B)** Distribution of exon sizes of lncRNA and protein-coding genes. **(C)** Transcript length comparison of lncRNAs and protein-coding genes. On average, lncRNAs have longer transcripts than protein-coding genes.

Strand-specific RNA-seq was finished *via* NovaSeq 6000, Illumina. Raw data on RNA-seq were submitted to the public database: National Genomics Data Center (https://ngdc.cncb.ac.cn/). The assigned accession of the submission is CRA009124. Then, 43,490,487 to 55,131,982 clean reads were obtained from a total of 94.73 G clean data (Supplementary Table S3). Mapping these reads to an updated version genome of SSB (Ma et al., 2020), the highest alignment rate of 91.85% was yielded. An average of 121,998 transcripts was assembled in each sample from aligned reads. Finally, a total of 40,573 merged transcripts were determined (Supplementary Table S3). Twenty-two transcripts shorter than 200 nT were first removed from 40,573 merged transcripts (Figure 1A). Then, 16,284 transcripts which contained single exons were also discarded. Up to 24,267 transcripts with multiple exons were filtered by CPAT, Swiss-Prot, and Pfam databases to exclude the possibility of protein-coding genes. Finally, a total of 3470 lncRNAs were discovered and characterized in SSB after removing small non-coding RNAs *via* NONCODE and Rfam databases. These lncRNAs were further divided into four categories, namely, intronic lncRNAs, lincRNAs, sense lncRNAs, and antisense lncRNAs, according to their relative positions and orientations on the genome (Figure 1B). Most lncRNAs (1879, 54.12%) were located in intergenic regions, the second-ranked sense lncRNAs (853, 24.57%) shared an overlap with the exon region of protein-coding genes, and 14.23% and 7.09% lncRNAs were antisense lncRNAs (493) and intronic lncRNAs (245) (Figure 1B). Among 3470 SSB lncRNAs, 2454 of which contained two exons, accounting for 70.68% (Figure 2A), and only 2.13% of lncRNAs contained seven or more exons. In contrast, only 13.27% protein-coding genes harbored two exons. In addition, the protein-coding genes with three exons accounted for the largest proportion (17.16%). The exons whose length was shorter than 200 bp made up for more than 70% in protein-coding genes, but there was less than 40% in exons shorter than 200 bp in lncRNAs (Figure 2B). As shown in Figure 2C, the average length of the SSB lncRNA transcript and the protein-coding genes were 1049 and 199 bp, respectively.

### Function analysis of differentially expressed lncRNA and adjacent protein genes

**FIGURE 3:**
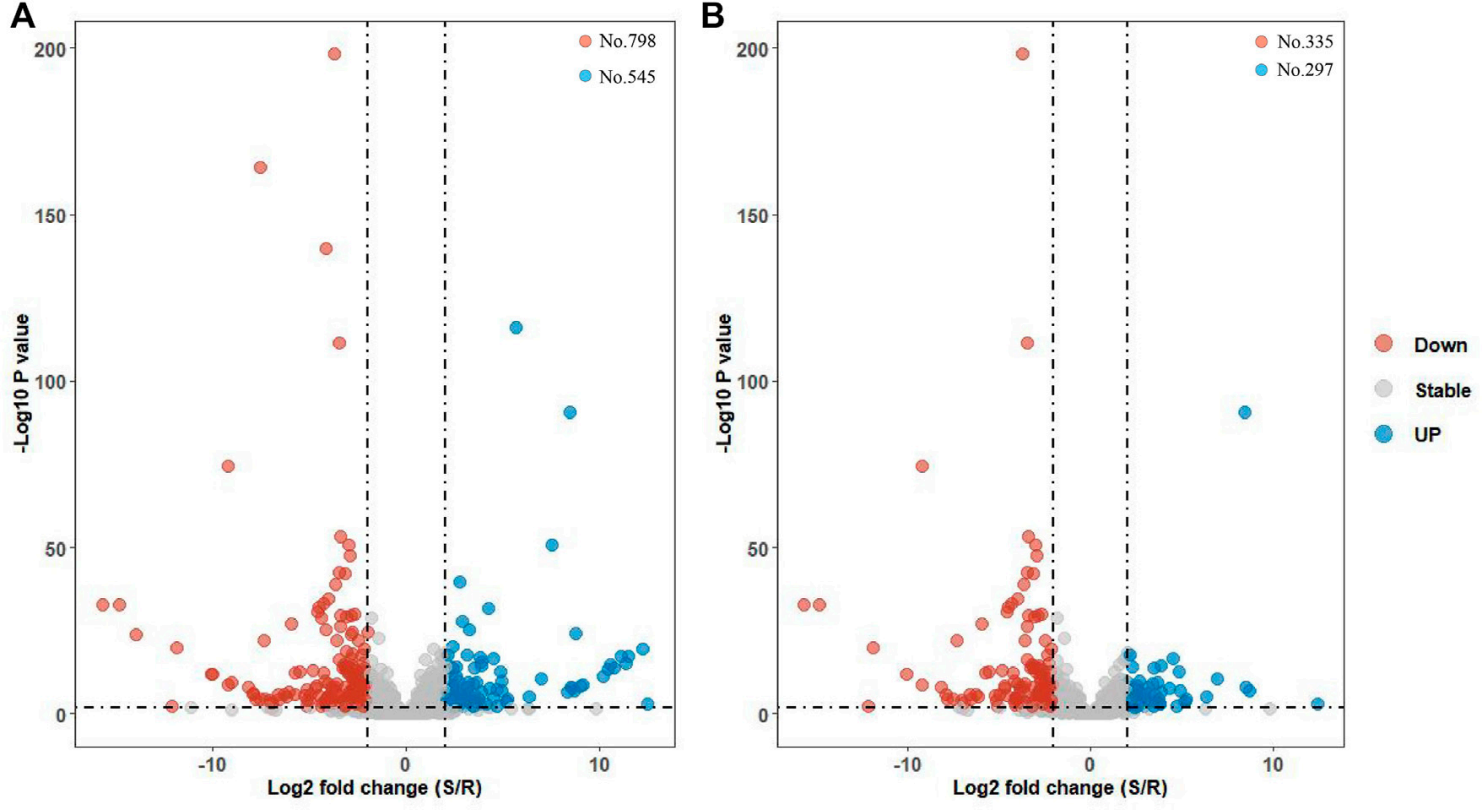
Volcano map of differentially expressed lncRNA genes. **(A)** Expression profile of all 1,343 dysregulated lncRNAs in S and R strains were plotted *via* the volcano map. **(B)** Expression profile of 632 DE lncRNAs in S and R strains. They met a strict screening criteria, that is, |log2 fold-change|>1, *p*-value < 01, and *p*-adj < 01.

Up to 3356 lncRNAs were gathered in S or R strains after removing 116 lncRNAs with FPKM=0 in all six libraries. There were 1343 lncRNAs dysregulated with |log2 fold-change|>1. Also, there were 798 and 545 lncRNAs highly expressed in R and S strains, respectively (Figure 3A). Moreover, a total of 632 differentially expressed (DE) lncRNAs met strict screening criteria, that is, *p*-value < 01, *p*-adj < 01, and |log2 fold-change|>1 (Figure 3B).There were 297 and 335 lncRNAs upregulated in S and R strains. Among these lncRNAs, 365, 31, 141, and 95 belonged to lincRNAs, intronic lncRNAs, sense lncRNAs, and antisense lncRNAs, respectively.

**FIGURE 4:**
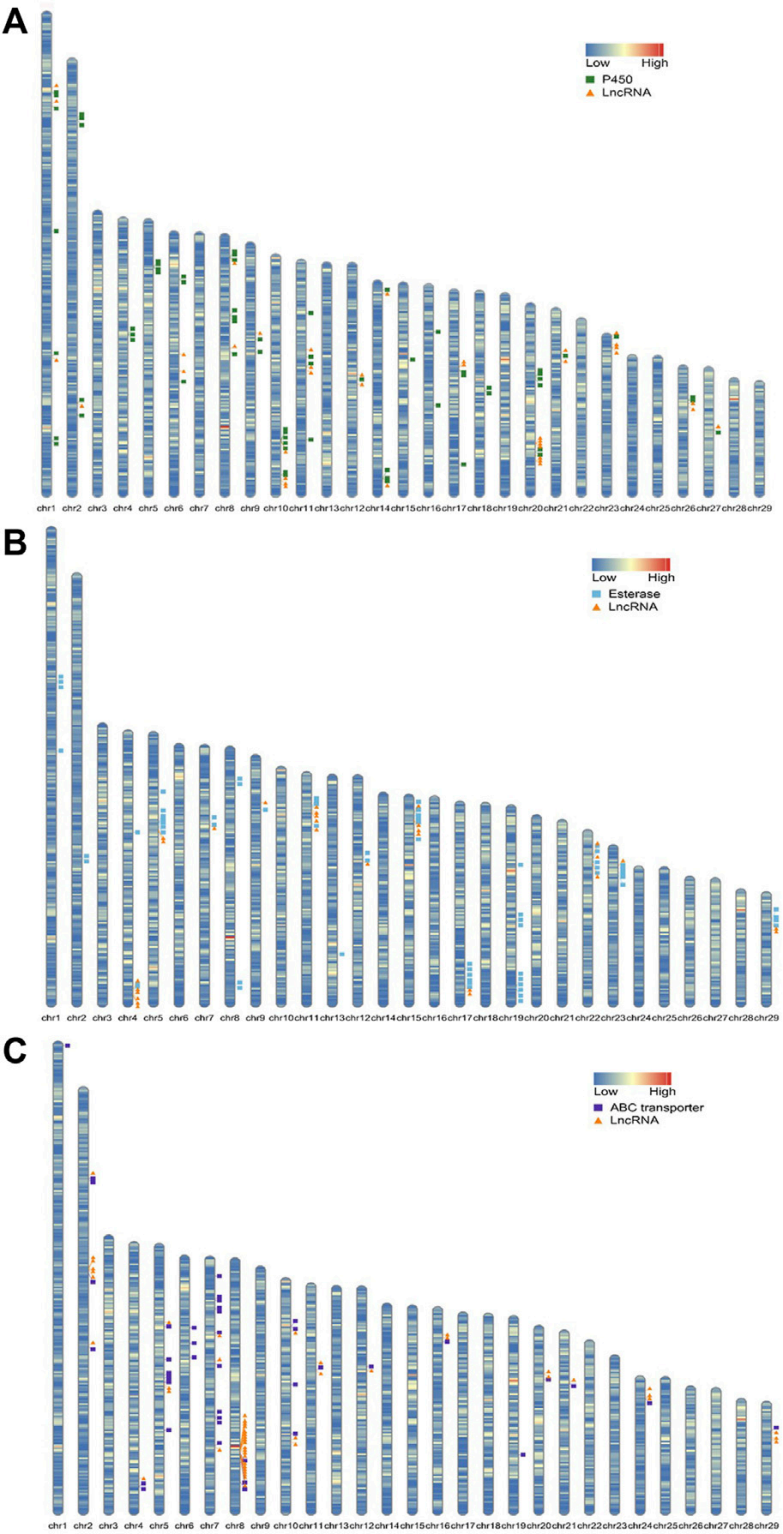
Chromosome maps showing the location of genes related to detoxification metabolic enzymes and dysregulated lncRNAs which were adjacent within 1000 kb. **(A)** P450 genes and dysregulated lncRNAs. **(B)** Esterase genes and dysregulated lncRNAs. **(C)** ABC genes and dysregulated lncRNAs.

In the detoxification enzyme gene family, P450s, carboxyl/cholinesterases/esterases, and ABC genes were related to the chlorantraniliprole resistance in SSB (Wang et al., 2015; Xu et al., 2019; Peng et al., 2021). Also, a 1000-kb range between the center of an lncRNA gene and a neighboring protein-coding gene transcription start site (TSS) was defined as a regulatory zone by the Genomic Regions Enrichment of Annotations Tool (GREAT) (McLean et al., 2010). To study the potential function of lncRNAs in chlorantraniliprole resistance, both the detoxification enzyme genes and DE lncRNAs were located in the genome of SSB. Sixty-six P450 genes were located on 20 different chromosomes (Figure 4A; Supplementary Table S4). Among the 38 lncRNAs adjacent to these P450 genes, the lincRNA, sense lncRNA, antisense lncRNA, and intronic lncRNA were 24, 7, 5, and 2, respectively. On chromosome10, the lincRNA *MSTRG.2776.2* was upregulated in the S strain as 8.45 log2 fold change (log2FC) as in the R strain, and it was 944, 528 bp apart from CYP9A12. For esterases, there were 67 carboxyl/choline esterases located on 16 chromosomes (Figure 4B; Supplementary Table S5), and a total of 27 lncRNAs were located nearby. On chromosome 9, the lincRNA *MSTRG.25727.1* was upregulated in the R strain as 6.54 log2FC as in the S strain, and CsuEst35 antennal esterase was 887, 454 bp apart from it. Forty-three of the 47 ABC transporter genes previously reported were successfully located in the genome of SSB. They are distributed on 16 different chromosomes (Figure 4C; Supplementary Table S6). At the same time, 51 lncRNAs were adjacent to them, including 31 lincRNAs, 12 sense lncRNAs, 6 antisense lncRNAs, and 2 intronic lncRNAs. ABCB4, D3, and G12 were located on chromosome 8, and the expression of D3 was upregulated and that of G12 was downregulated in the R strain (Peng et al., 2021). A total of 18 lncRNAs were dysregulated in the R strain (Figure 4C; Supplementary Table S6) as lincRNAs *MSTRG.25315.3* and *MSTRG.25316.8* were upregulated.

### Differentially expressed long intergenic non-coding RNAs

Considering lincRNAs, also known as long intergenic non-coding RNAs, accounted for 54.10% of the number of lncRNAs (Figure 1B, 1879 out of 3470), our focused analyses revealed there were 167 and 198 lincRNAs differentially highly expressed in S and R strains, respectively. Those lincRNAs whose value of |log2FC| ranked in the top 20 were chosen in the following analysis (Tables 1, 2). The log2FC of lincRNA *MSTRG.6875.40* was 12.44, ranking it the highest in the top in the S strain (Table 1), whereas *MSTRG.25315.3* was 15.70, ranked first in the R strain (Table 2). The log2FC values of differentially expressed *MSTRG.22483.6* and *MSTRG.13055.1* were 3.82 and 6.16, as they were listed last. These 40 lincRNAs were distributed on 17 different chromosomes (Chr1, 2, 4, 6, 8, 9, 10, 12, 13, 15, 16, 17, 18, 21, 26, 27, and 28) or scaffolds (scaffold 789 and scaffold 83). The length of them varied widely, ranging from 231 to 11,712 bp. The exon numbers varied from 2 to 7. For example, there were seven, six, and five exons in *MSTRG.25315.3*, *MSTRG.11012.32* (Table 2), and *MSTRG.6875.40* (Table 1), respectively.

**TABLE 1:** Top 20 predictive lincRNAs differential highly expressed in the S strain.

LncRNA ID	Chromosome strand	Exon number	Locus	Length (bp)	Log2 fold change (SvsR)	*p*-value	P-adj
MSTRG.6875.40	Chr15−^a^	5	10767271-10770633;10770728-10775914; 10776269-10776550;10776821-10778416; 10778901-10779037	10565	12.44	0.00032639	0.001405702
MSTRG.3932.19	Chr12−	4	1060573-1060629;1062299-1062828; 1063040-1063243;1063448-1065307	2651	8.68	7.76E-09	8.36E-08
MSTRG.11012.44	Chr2+^b^	5	17944191-17945059;17946907-17947142; 17950697-17950970;17953572-17954029; 17955731-17956328	2435	8.50	6.46E-10	8.17E-09
MSTRG.2776.2	Chr10−	2	26454062-26455136;26475255-26475998	1819	8.45	2.44E-94	2.03E-91
MSTRG.20713.2	Chr4+	2	21929023-21932131;21932153-21934797	5754	7.84	1.10E-05	6.58E-05
MSTRG.18071.3	Chr28+	2	10375469-10380019;10380114-10380217	4655	7.61	0.0001034	0.000501427
MSTRG.3932.24	Chr12−	4	1061852-1061880;1062299-1063243; 1063448-1063651;1063849-1065581	2911	6.92	1.48E-12	2.60E-11
MSTRG.3932.15	Chr12−	4	1060572-1060629;1062299-1062646; 1062829-1063243;1063448-1065307	2681	6.33	1.10E-06	8.07E-06
MSTRG.17405.1	Chr27+	2	11469445-11470705;11472274-11472518	1506	5.21	4.23E-06	2.78E-05
MSTRG.10602.2	Chr2+	2	4355379-4355881;4407450-4407698	752	5.14	0.00111817	0.004155295
MSTRG.4909.2	Chr13+	2	1575626-1585474;1585507-1585531	9874	5.14	3.31E-05	0.000180403
MSTRG.17825.3	Chr28−	3	3751409-3767991;3786614-3786676; 3816087-3816305	16865	5.14	3.88E-05	0.000208446
MSTRG.7482.1	Chr16−	2	3114234-3114956;3114982-3115061	803	4.92	8.31E-09	8.92E-08
MSTRG.29804.10	Scaffold 789−^c^	5	37879-38503;38652-38681;38817-38878; 38902-38943;42553-43887	2094	4.84	1.32E-14	2.96E-13
MSTRG.29873.1	Scaffold 83−	2	21,148-22080;22110-23497	2321	4.69	0.00181057	.00631492
MSTRG.17825.2	Chr28−	4	3751409-3758714;3758762-3767991; 3809101-3809293;3816087-3816347	16990	4.51	4.84E-19	1.83E-17
MSTRG.9533.1	Chr18−	2	22324962-22326781;22332655-22333456	2622	4.32	3.06E-09	3.49E-08
MSTRG.29805.1	Scaffold 789+	2	37827-38367;42417-42970	1095	3.89	2.19E-08	2.19E-07
MSTRG.713.1	Chr1+	2	19868296-19869308;19870259-19876056	6811	3.88	1.54E-16	4.42E-15
MSTRG.22483.6	Chr6+	2	2938722-2938846;2941360-2944153	2919	3.82	0.00036102	0.001534732

^a^ The short minus indicated the predicted lncRNA to be located in the reverse complementary strand of the assembled chromosome.

^b^ The short plus indicated the lncRNA to be located in the sense strand of the chromosome.

^c^ When assembling, large fragments were not successfully assembled onto one chromosome. Some lncRNAs are located on large fragments.

**TABLE 2:** Top 20 predictive lincRNAs differential highly expressed in the R strain.

LncRNA ID	Chromosome strand	Exon number	Locus	Length (bp)	Log2 fold change (R *vs*. S)	*p*-value	P-adj
MSTRG.25315.3	Chr8+^**a**^	7	22677723-22678101;22749104-22749274; 22749522-22749632;22749844-22749990; 22750410-22750610;22750917-22751064; 22751107-22756133	6184	15.70	1.39E-35	1.64E-33
MSTRG.25316.8	Chr8-^**b**^	2	22699728-22709465;22714458-22716431	11,712	14.85	2.02E-35	2.37E-33
MSTRG.25723.1	Chr9-	2	5260089-5260324;5260848-5261965	1354	12.11	0.001931984	.006687415
MSTRG.17282.3	Chr27-	2	9094064-9096939;9097421-9098545	4001	11.85	2.80E-22	1.47E-20
MSTRG.11012.32	Chr2+	6	17944191-17944758;17946606-17946660; 17951139-17951384;17952309-17953058; 17953983-17954029;17955731-17956328	2264	10.05	7.69E-14	1.56E-12
MSTRG.17788.5	Chr28+	2	3055427-3055865;3067332-3067769	877	9.23	6.90E-78	4.02E-75
MSTRG.7200.3	Chr15-	2	22250366-22253327;22253357-22254342	3948	9.21	1.25E-10	1.74E-09
MSTRG.17632.2	Chr28+	2	155617-156240;157354-157412	683	8.17	6.66E-10	8.41E-09
MSTRG.29804.8	Scaffold 789.-^**c**^	5	37827-38499;38648-38681;38817-38943; 42553-42688;42828-43181	1324	7.92	1.75E-07	1.48E-06
MSTRG.11011.14	Chr2-	6	17944162-17945020;17946868-17946941; 17950424-17950483;17950556-17951345; 17952270-17952464;17956158-17956328	2149	7.81	7.04E-06	4.39E-05
MSTRG.8464.1	Chr17+	2	13024071-13024252;13024645-13024942	480	7.54	6.81E-06	4.26E-05
MSTRG.11011.22	Chr2-	5	17944162-17945790;17949556-17949599; 17953157-17953625;17954550-17955319; 17956097-17956328	3144	7.34	2.29E-24	1.43E-22
MSTRG.16430.2	Chr26+	2	6123733-6123812;6127962-6128560	679	7.05	1.20E-05	7.14E-05
MSTRG.16712.6	Chr26+	3	12454424-12454523;12454611-12454620; 12471577-12471779	313	6.93	0.000146114	.00068547
MSTRG.8465.1	Chr17+	2	13034463-13034697;13035057-13035745	924	6.62	1.12E-07	9.79E-07
MSTRG.25727.1	Chr9-	4	5596254-5596306;5596605-5596722; 5596854-5596918;5597006-5597106	337	6.54	4.62E-06	3.00E-05
MSTRG.3908.1	Chr12-	2	866882-866923;875029-875217	231	6.52	5.89E-05	.000303147
MSTRG.3932.9	Chr12-	5	1060001-1060089;1061991-1062027; 1062647-1062828;1063040-1063243; 1064053-1065319	1779	6.44	0.00024098	.001070053
MSTRG.8218.1	Chr17-	2	3839813-3840316;3846632-3848147	2020	6.30	1.32E-07	1.14E-06
MSTRG.13055.1	Chr21+	2	3668903-3668944;3674496-3675310	857	6.16	8.12E-07	6.10E-06

^a^ The short plus indicated the lncRNA to be located in the sense strand of chromosome.

^b^ The short minus indicated the predicted lncRNA to be located in the reverse complementary strand of the assembled chromosome.

^c^ When assembling, large fragments were not successfully assembled onto one chromosome. Some lncRNAs are located on large fragments.

**FIGURE 5:**
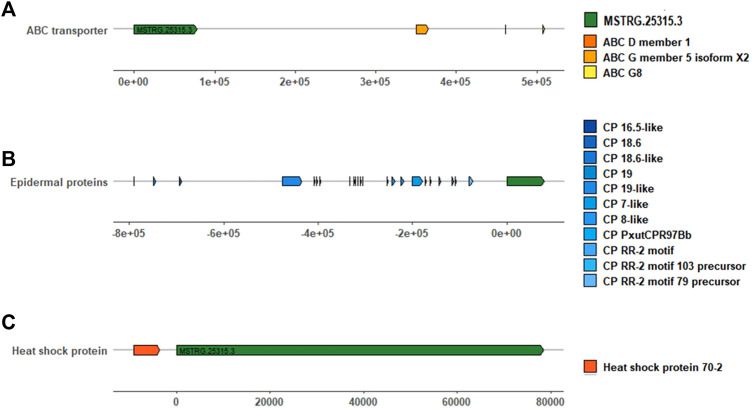
LincRNA *MSTRG.25315.3* and its adjacent protein-coding genes involved in pesticide resistance within 1000 kb. **(A)** Green square represents *MSTRG.25315.3*, and orange, brownish red, and yellow squares represent ABC genes. **(B)** Gradient blue squares represent different cuticle protein genes. **(C)** Red square represents the heat shock protein gene. The long solid line and the number marked above represent the distance (bp) between them, *MSTRG.25315.3* and adjacent genes.

Protein-coding genes within 1000 kb adjacent to lincRNA were collected. *MSTRG.25315.3* was the top differentially upregulated gene in the R strain (Table 2, 15.70 of log2FC) with 83 coding genes located. Heat shock protein 70–2 (GenBank accession: AGR84224.1) was only 1,770 bp close to *MSTRG.25315.3* (Figure 5). The lincRNA *MSTRG.25727.1* was 337 bp long, but it harbored four exons and was elevated to a log2FC value of 6.54 in the R strain compared to the S strain. Among 20 mRNA genes nearby, antennal esterase was 887, 454 bp adjacent to *MSTRG.25727.1*. In addition, sodium channel protein was 429, 858 bp adjacent to *MSTRG.7200.3* (Table 2).

With regards to the top 20 highly expressed lincRNAs in the S strain, *MSTRG.2776.2* was 961, 794 bp adjacent to cytochrome 9A20 (annotated from *Bombyx mori*, GenBank accession: BAI47532.1). Sixty-four protein-coding genes were close to *MSTRG.6875.40* within 1000 kb, and UDP-glucuronosyl transferase 2B31-like was 990, 115 bp apart from it. *MSTRG.20713.2* was upregulated in the S strain (Table 1, 7.84 of log2FC), and it was 494, 273 bp away from 4A1-like gene, which is a family member of solute carrier organic anion transporters, encoding membrane proteins that are involved in solute (charged and uncharged organic molecules, and inorganic ions) transportation. They are likely to be involved in pesticide metabolism and transport.

### Validation of DE lincRNAs in two strains by RT-PCR and qRT-PCR

**FIGURE 6:**
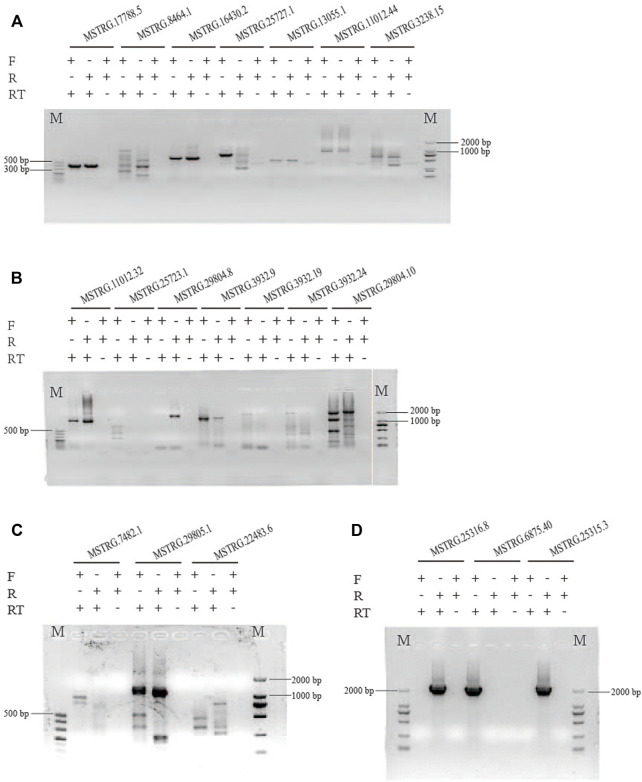
Strand-specific PCR of 20 randomly selected lincRNAs to determine transcription orientation. The different electrophoretic bands of lncRNAs with similar PCR product size were shown in **(A–D)**. The results indicated that 13 lincRNAs are strand-specific, that is, five came out as forward-stranded (*MSTRG.25723.1*, *3932.19*, *3932.24*, *7482.1*, and *6875.40*) and eight were reverse-stranded (*MSTRG.8464.1*, *25727.1*, *11012.32*, *29804.8*, *29804.10*, *22483.6*, *25315.3*, and *25316.8*. The other seven lincRNAs can be amplified by both primers.

We randomly selected 20 lincRNA candidates shown in Tables 1, 2 to be validated by strand-specific RT-PCR. Results showed 13 lincRNAs were strand-specific, that is, five came out forward stranded (*MSTRG.25723.1*, *3932.19*, *3932.24*, *7482.1*, and *6875.40*) and eight were reverse stranded (*MSTRG.8464.1*, *25727.1*, *11012.32*, *29804.8*, *29804.10*, *22483.6*, *25315.3*, and *25316.8*) (Figure 6). Of the 13 strand-specific lincRNAs, only four were in the same direction as the originally labeled strand (Tables 1, 2, *MSTRG.25316.8*, *29804.8*, *25727.1*, and *29804.10*). The other seven lincRNAs can be amplified by both primers (Figure 6).

**FIGURE 7:**
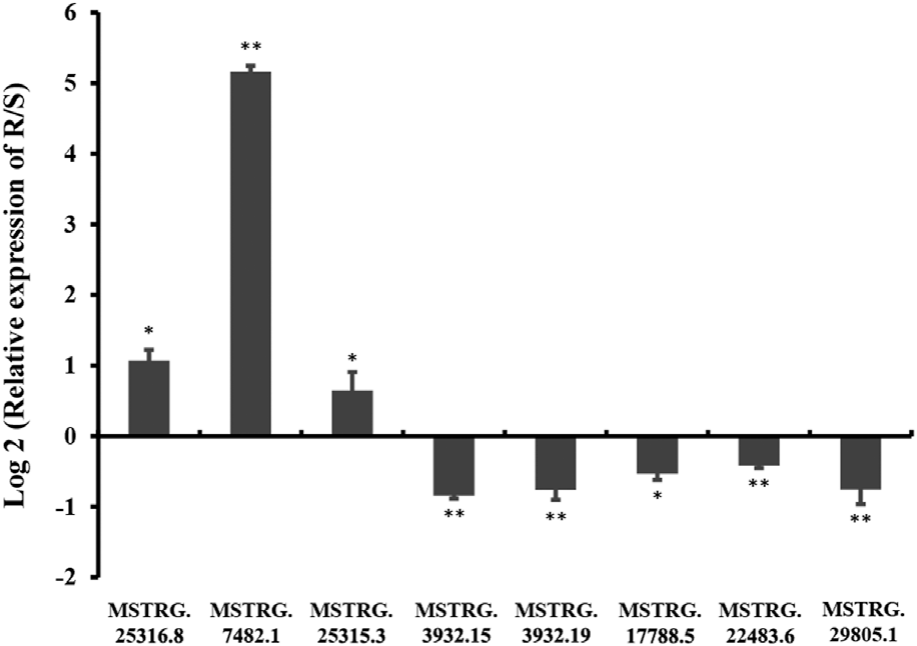
Relative expression levels of lincRNAs in chlorantraniliprole-resistant strains (R) and susceptible strains (S).The column represents the value of log2 (relative expression levels of lincRNAs in the R strain compared to the S strain). Each column represents the mean of three to four biological samples. Error bars represent the standard deviation from the mean. The change in the expression level was calculated using the 2^−ΔΔCt^ method. Data were normalized to the expression of housekeeping genes (Actin A1). Asterisks on the error bars show significant differences (*p* < 05 or *p* < 01).

To verify the RNA-seq results, the relative expression levels of three randomly selected DE lincRNAs in the R strain (*MSTRG.25315.3*, *MSTRG.25316.8*, and *MSTRG.17788.5*) and five lincRNAs in the S strain (*MSTRG.3932.19*, *MSTRG.3932.15*, *MSTRG.7482.1*, *MSTRG.29805.1*, and *MSTRG.22483.6*) were examined by qRT-PCR (Figure 7). Consistent results were observed between the qRT-PCR results of most of the selected strains and the sequencing data except for *MSTRG.17788.5* and *MSTRG.7482.1*. The two lincRNAs displayed the opposite trend. The expression of *MSTRG.17788.5* was 9.23 times the log2FC value in the R strain as that in the S strain (Table 2), but it turned out 0.69 times in the R strain as that in the S strain. On the contrary, the expression of *MSTRG.7482.1* was first 4.92 of log2FC in the S strain as that in the R strain (Table 1), but it turned out 35.86 times in the R strain confirmed by qRT-PCR.

### Functional analysis of the lincRNA MSTRG.7482.1

**FIGURE 8:**
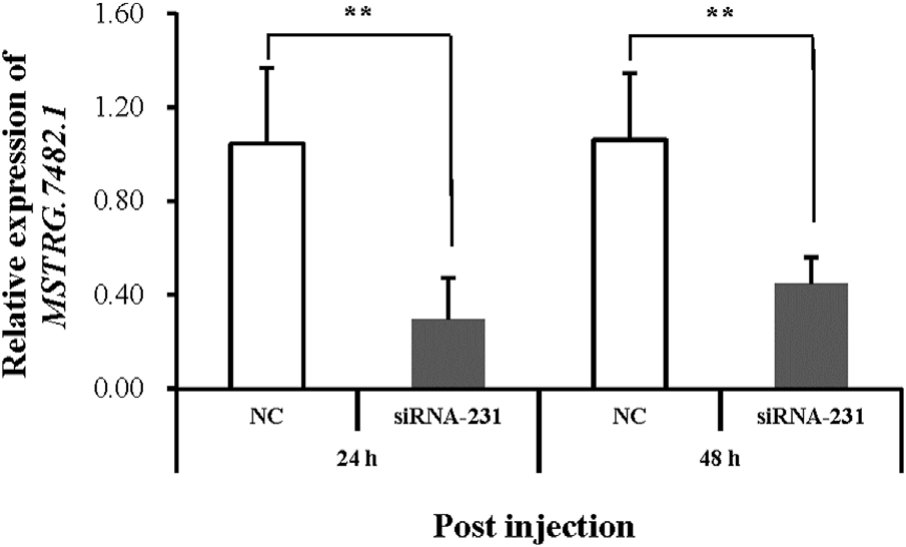
Relative expression of *MSTRG.7482.1* after injection with siRNA. Asterisks on the error bars show significant differences (*p* < 01).

*MSTRG.7482.1* had the highest differential expression folder change among the three lincRNAs, which is highly expressed in the R strain (Figure 7). We first used qRT-PCR to examine the expression of *MSTRG.7482.1* in different stages of development, like eggs, each instar larvae, pupae, and male and female moths (Supplementary Figure S1). Altogether, *MSTRG.7482.1* expressed highly from eggs until the sixth-instar larvae. Then, it declined sharply in the pupal stage and was expressed to the lowest in the female moth. The expression profile suggested *MSTRG.7482.1* functions mainly in larvae, which is, the feeding stage. The siRNA was designed from position 231 based on the sequence of *MSTRG.7482.1*, and we successfully knocked down *MSTRG.7482.1* gene by injecting siRNA in the fourth-instar larvae (Figure 8). The expression of *MSTRG.7482.1* was knocked to about 40% compared to control in both 24 and 48 h after injection.

**FIGURE 9:**
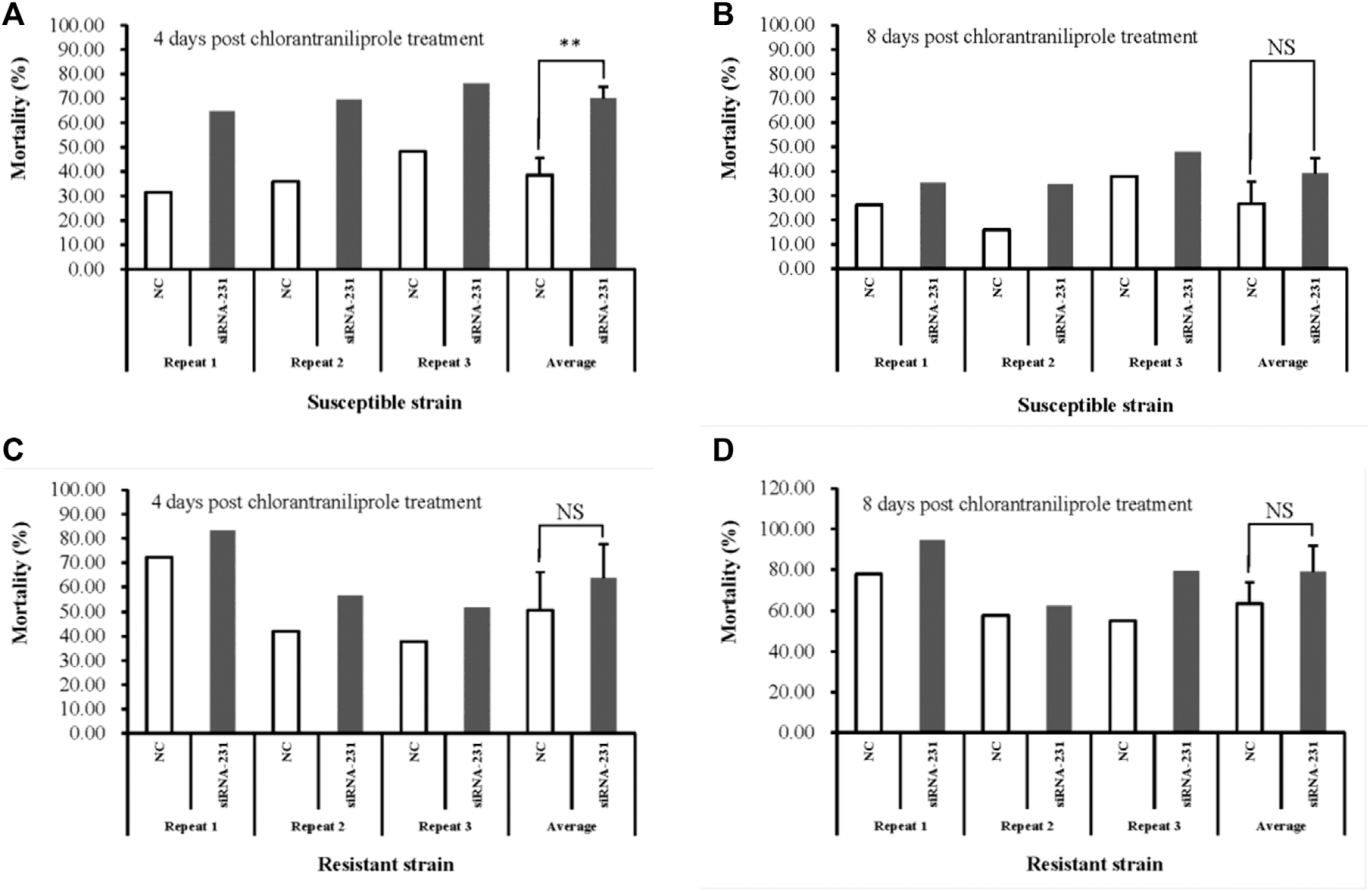
Susceptibility to chlorantraniliprole after silencing *MSTRG.7482.1* with siRNA. The toxicity assay was conducted in both the S strain **(A,B)** and R strain **(C,D)**. Mortality was calculated 96 h after chlorantraniliprole exposure. All experiments were conducted in triplicate. Asterisks on the error bars show significant differences (*p* < 01). NS on the error bars, however, show no significant differences.

To detect the effect of the downregulated *MSTRG.7482.1* gene on chlorantraniliprole sensitivity, the fourth-instar larvae injected with siRNA-231 were removed onto an artificial diet with chlorantraniliprole treatment, according to the LC_50_ dose in the S or R strain for 96 h, respectively. In the S strain, the RNAi group showed 70.09% average mortality, which is significantly higher than that of control (mortality: 38.62%) four days after pesticide treatment (Figure 9A). On day 8 post-chlorantraniliprole treatment, both mortality of siRNA-231 and the control group declined and it turned out closer (39.36% *vs*. 26.75%) (Figure 9B). In the R strain, the average mortality of the siRNA-231 group was higher than that of the control at 4 and 8 days after pesticide treatment; there was no statistically significant difference (Figures 9C, D).

## Discussion

LncRNA has attracted much attention due to its unique features and molecular mechanisms involved in variety of biological functions (Wang and Chang 2011; Quinn and Chang 2016). In *Drosophila*, lncRNAs are responsible for the regulation of development (such as neuromuscular junction, embryo, and organ), behavior (sheep, mating, courtship, and locomotion), sex control, and dosage compensation. In addition, lncRNAs also help fruit flies against some stressors, including heat, bacterial infection, and wasp attacks (Li et al., 2019). As the sequencing technology advances, much more lncRNAs have been discovered in other non-model insects. A total of 2,914 lncRNAs were identified in *Aphis citricidus*, and down-regulation of *Ac_lnc54106.1* resulted in malformed wings, which showed lncRNAs mediated the wing plasticity (Shang et al., 2021). 20-Hydroxyecdysone (20E) was injected into the hemolymph of silkworms to study autophagy. Predictive 6,493 cis pairs and 42,797 trans pairs of lncRNA–mRNA were found, and functional analysis of *LNC_000560* suggested it potentially regulated *Atg4B* and participated in the 20E-induced autophagy of the fat body (Qiao et al., 2021). In addition, lncRNAs might have key roles in conferring insecticide resistance and regulating the metamorphosis development in *P. xylostella* (Liu et al., 2017). Unfortunately, the molecular mechanism of how lncRNAs regulate pesticide resistance of SSB is still unclear.

SSB has developed severe resistance to chlorantraniliprole (Sun et al., 2018; Huang et al., 2020). Here, a total of 3,470 lncRNAs were identified at last from six strand-specific libraries in both S and R strains. The total number of lncRNAs was smaller than 6,171 reported in *B. dorsalis* (Meng et al., 2021) and 11,978 lncRNAs in *S. litura* (Shi et al., 2022), but it was greater than that reported in *P. xylostella* (Zhu et al., 2017). Interestingly, the number of lncRNAs was different even in the same species. In *P. xylostella*, 3,324 lncRNAs were found from 13 RNA-seq datasets, 1,309 lncRNAs were identified in 9 libraries, and 3,844 lincRNAs were discovered based on 7 RNA sequencing libraries (Etebari et al., 2015; Liu et al., 2017; Zhu et al., 2017). The difference in quantity may come from many factors, such as different methods of library construction, different tissues obtained from samples, and different insecticide exposure. There were 10 kinds of classification for lncRNAs, according to different criteria in the early stage (St Laurent et al., 2015). For example, lncRNA was divided into six types, that is, convergent, divergent, overlapping, intergenic, enhancer, host of miRNA, and intronic in *Drosophila* (Li et al., 2019). At present, much research refers to the location of lncRNAs to classify them into four categories, including intronic lncRNAs, lincRNAs, sense lncRNAs, and antisense lncRNAs (Zhu et al., 2017; Meng et al., 2021; Yang et al., 2021). Although the composition proportion of intronic lncRNA, sense, and antisense lncRNA is different, the proportion of lincRNA is the highest. This is also reflected in SSB, where lincRNA accounts for 54.10% of lncRNA (1879 in 3470). Up to 70.68% SSB lncRNAs harbored two exons, which was similar to that in *N. lugens* (77.9%) (Xiao et al., 2015), *P. xylostella* (74.49%) (Liu et al., 2017), and *B. dorsalis* (84.36%) (Meng et al., 2021). Usually, the average transcript length of lncRNAs is shorter than protein-coding genes. The average lengths of the *P. xylostella* lncRNA transcript and protein-coding genes were 912 bp and 1,385 bp, respectively (Liu et al., 2017). However, the average transcript length of SSB lncRNAs (1049 bp) was much longer than that of protein-coding genes (199 bp). This may be a result of species specificity.

The association between lncRNAs and adjacent genes is one of many methods to study the regulatory function of lncRNAs. However, the distance between lncRNA and its neighbor genes varied. In *N. lugens*, adjacent protein-coding genes less than 5 kb apart from lncRNAs were statistically significant than randomly selected coding genes (Xiao et al., 2015). Zhu *et al.* predicted cis regulation of many protein-coding genes, which were found within a 10-kb range upstream or downstream from the target lncRNAs (Zhu et al., 2017). In *B. dorsalis*, 793 target genes were predicted *via* searching 100 kb upstream and downstream (Meng et al., 2021).To more comprehensively understand the association between lncRNA and potential target genes, we set the distance between the protein-coding gene and lncRNA within 1000 kb (McLean et al., 2010; Mitchell et al., 2017). It is well known that RyR is the target of chlorantraniliprole, and a previous report attempted to study the relation of chlorantraniliprole resistance with lncRNA and RyR (Zhu et al., 2017). Two co-expressed lncRNAs with RyR, TCONS_00013329 and TCONS_00056155, were found in *P. xylostella*. In SSB, there was no RyR found within 1000 kb of differentially expressed lincRNAs in this region. This might be related to the dataset used in the analysis that we focused on lincRNAs rather than all the four types of lncRNAs. Other three types of lncRNAs will be investigated in the future research and is beyond the scope of this study.

Fortunately, there were meaningful findings in eight lincRNAs that had been verified by qRT-PCR. *MSTRG.25316.8*, *MSTRG.25315.3*, and *MSTRG.7482.1* were highly expressed in the R strain. Among 83 adjacent genes near to *MSTRG.25315.3*, *hsp70-2* (GenBank accession: AGR84224.1) was only 1,770 bp nearby. Exposed to carbaryl, three small-molecule heat shock (smhsp) genes (*hsp20. 3*, *hsp19. 1*, and *hsp17. 0*) were upregulated in *Lymantria dispar* to varying degrees (Peng et al., 2017). These results suggested *hsp* may be involved in responding to the pesticide stress. In addition, three ATP-binding cassette sub-family genes G5, D3, and G8 were located in 310, 421, and 468 kb. In a previous study, *CsABCG5* and *G8* were significantly highly expressed in the resistant strain of SSB against chlorantraniliprole (Peng et al., 2021).

The expression of *MSTRG.7482.1* in the R strain was 35.86 times as that in the S strain (Figure 7), and RNAi assay showed that knock-down of *MSTRG.7482.1* resulted in an increase of average mortality by 31.47% (Figure 9A). However, such a statistic significant phenomenon was only observed at four days post-pesticide treatment in the S strain. In the test of the R strain, the mortality of the treatment group in all groups was higher than that of the control group to varying degrees, but it was not statistically significant (*p*-value >.05). On the whole, although not all repeated experimental results were statistically significant, the mortality of the RNAi group is indeed higher than that of the control. In other words, RNAi of *MSTRG.7482.1* increased the sensitivity to chlorantraniliprole. In *S. litura*, RNAi of *LNC_004867* or *LNC_006576* increased the mortality from 14.68% to 34.69% against indoxacarb (Shi et al., 2022). Because *LNC_006576* and *LNC_004867* were significantly expressed in the developmental stages of 1-6-instar larvae, it suggested the two lncRNAs may play important roles in the metabolism of insecticides or plant chemicals in *S. litura*. In SSB, the lincRNA *MSTRG.7482.1* was also expressed highly across the feeding larval stage (Fig. S1), and it might be associated with xenobiotic detoxification. A total of 35 adjacent protein-coding genes within 1000 kb apart from lincRNA *MSTRG.7482.1* were searched (Supplementary Table S7). There were 14 undefined protein residues. Among the other 21 genes, indole-3-acetaldehyde oxidase-like isoform X1 (*Bombyx mandarina*) was the nearest which is 85, 966bp to *MSTRG.7482.1*. Alpha-(1, 6)-fucosyltransferase (*Galleria mellonella*) plays an important role in cell recognition, proliferation, and metabolic activities. At a distance of 433, 455 bp, there was cuticle secretory protein (*Ostrinia furnacalis*). However, the function of these adjacent genes and their association with resistance mechanisms are unknown. In future, more precise target prediction and carefully functional studies are needed to elucidate the relation to the mechanism of chlorantraniliprole resistance.

## Conclusion

In this study, a total of 3,470 lncRNAs were identified from six RNA-seq libraries of SSB, including 1,879 intergenic lncRNAs, 245 intronic lncRNAs, 853 sense lncRNAs, and 493 antisense lncRNAs. In addition, 632 DE lncRNAs were discovered. Adjacent protein-coding genes of each of the 20 top DE lincRNAs in both S and R stains were analyzed. Strand-specific RT-PCR was conducted to determine the transcript orientation of 20 randomly selected lincRNAs. qRT-PCR was performed to verify the expression level of eight lincRNAs, including *MSTRG.25315.3* and *MSTRG.7482.1*. RNAi and bioassay analyses indicated that *MSTRG.7482.1* was involved in chlorantraniliprole resistance. All the results provide the basis for better understanding about the roles of lncRNAs in regulating the resistance of chlorantraniliprole and other insecticides in SSB.

## Data Availability

The datasets presented in this study can be found in online repositories. The names of the repository/repositories and accession number(s) can be found below: https://ngdc.cncb.ac.cn/, CRA009124.

## Supplementary material

Supplementary Figure S1 Expression profile of MSTRG.7482.1 in different stages across life time in the S strain. Each column represents the mean of three biological samples. Error bars represent the standard deviation from the mean. The change in the expression level was calculated using the 2^-ΔΔCt^ method. Data were normalized to the expression of housekeeping genes (Actin A1). Different letters on the error bars show significant differences (*p* < .05).
