# Supplementary material for: Genome-wide identification and functional analysis of long non-coding RNAs in Chilo suppressalis reveal their potential roles in chlorantraniliprole resistance
Source: Front Physiol. 2023 Jan 9;13:1091232. doi: 10.3389/fphys.2022.1091232 (PMC9868556; doi:10.3389/fphys.2022.1091232)
Supplement: Supplementary file 1 [file Table1.DOCX]

**Table S1.** Primers used for validation of reverse transcription PCR.

| LincRNA ID | Forward primer (sense) (5’——3’) | Reverse primer (antisense) (5’——3’) | Product length (bp) |
| --- | --- | --- | --- |
| MSTRG.25315.3 | ACGGATTGTTTTCCTTCTTTAAT | ATGTATTGAAATGCACTGGAACT | 2144 |
| MSTRG.25316.8 | TACCGAGTTTATTTAAAGGAAGCA | TCAAGAGATAACTTAGCCAGAGCT | 2133 |
| MSTRG.25723.1 | CATGTTACCGTTAATACCTT | ATTTGTAAGATTTTACCCCT | 723 |
| MSTRG.11012.32 | CAAATCTCCAATTACCACCGT | TCGACAGTTTCGGTAGGCTTG | 1036 |
| MSTRG.17788.5 | GGTCCTGCGAGTGCCCGAAA | CCTCGGCGTACGTGCGTCTC | 397 |
| MSTRG.29804.8 | TAATATCTTGTTCACTGCGGTT | CTCAATAAAACTTCTAACGCAAC | 1721 |
| MSTRG.8464.1 | ATCAAACAAAATGAAGTTCGC | AAATGTGACCCCAATATCAGA | 414 |
| MSTRG.16430.2 | AAACCCCTGGTGTTCGGTA | ACTTTATAGGGCCATAGAGCTG | 633 |
| MSTRG.25727.1 | CCTCATGTGATGGCGCAGCCT | TGGGACAGTATCCTTCGTGCCTCA | 262 |
| MSTRG.3932.9 | TCTGCAGCCACACATAGACT | CTCTCGTATGGGATTGTGAT | 1616 |
| MSTRG.13055.1 | TTGATATCGCAGGTAGTCAC | TTAAATATGATGCGAATGGTT | 711 |
| MSTRG.6875.40 | AGTTACTCTTGTATGATGTCCCA | GTAGCATAAAACTAGCGATCTCCA | 2033 |
| MSTRG.3932.19 | ATCTTGGCTTAAATGAGGGTC | GCAATACAAACAGGCTGCAT | 1964 |
| MSTRG.11012.44 | CGAAACGCCAGAGGGCAAGACA | TGCGCTTGGTCACCTTCTTCGT | 971 |
| MSTRG.3932.24 | CCTTGGATTCGCTACAGTCGAG | GCAATACAAACAGGCTGCAT | 2047 |
| MSTRG.3932.15 | GCGACTGAGACCAGGGCACC | CCACCACGGCTGCGCCTAAA | 748 |
| MSTRG.7482.1 | ATCACGTGTAATTGTTTTGGTC | TCAGATATTTAGCAGCGGGTT | 740 |
| MSTRG.29804.10 | TCTCTTTTGGCTGATAGAACC | AAAACTTCTAACGCAACCTG | 1932 |
| MSTRG.29805.1 | ATAAAACTTCTAACGCAACCTG | ATGTAAGCCCTGAAATAGCC | 954 |
| MSTRG.22483.6 | GTTAGCAGACATCCACGGAA | ACAACTATAGAAAGATTGCCACT | 764 |
