# Supplementary material for: Genome-wide identification and functional analysis of long non-coding RNAs in Chilo suppressalis reveal their potential roles in chlorantraniliprole resistance
Source: Front Physiol. 2023 Jan 9;13:1091232. doi: 10.3389/fphys.2022.1091232 (PMC9868556; doi:10.3389/fphys.2022.1091232)
Supplement: Supplementary file 2 [file Table7.DOCX]

**Table S7.** Thirty-five protein-coding genes adjacent to lincRNA *MSTRG.7482.1* within 1000 kb.

| **Gene ID** | **Annotation based on Blastx to NCBI** | **Length (bp)** | **Distance to *MSTRG.7482.1* (bp)** |
| --- | --- | --- | --- |
| scaffold264.len756920.5 | XP_022113187.1 mitochondrial import inner membrane translocase subunit TIM44 [*Pieris rapae*] | 1311 | -977239 |
| scaffold264.len756920.4 | XP_026730496.1 echinoderm microtubule-associated protein-like 2 isoform X1 [*Trichoplusia ni*] | 2580 | -897368 |
| scaffold264.len756920.3 | XP_035437879.1 MD-2-related lipid-recognition protein-like [*Spodoptera frugiperda*] | 426 | -741630 |
| scaffold264.len756920.2 | RVE43287.1 hypothetical protein evm_012046 [*Chilo suppressalis*] | 546 | -701526 |
| scaffold264.len756920.1 | XP_013173006.1 PREDICTED: uncharacterized protein LOC106121753 isoform X3 [*Papilio xuthus*] | 3444 | -652206 |
| scaffold1.len11383612.130 | XP_013196714.1 PREDICTED: neuroglobin-like [*Amyelois transitella*] | 582 | -437563 |
| scaffold1.len11383612.131 | RVE55233.1 hypothetical protein evm_000131 [*Chilo suppressalis*] | 432 | -292909 |
| scaffold295.len631900.20 | CAB3245138.1 unnamed protein product [*Arctia plantaginis*] | 528 | -29080 |
| scaffold295.len631900.19 | XP_028031987.1 indole-3-acetaldehyde oxidase-like isoform X1 [*Bombyx mandarina*] | 2469 | -85966 |
| scaffold295.len631900.18 | XP_035437504.1 symplekin-like isoform X2 [*Spodoptera frugiperda*] | 3528 | -133298 |
| scaffold295.len631900.17 | XP_028031978.1 uncharacterized protein LOC114244386 [*Bombyx mandarina*] | 567 | -178331 |
| scaffold295.len631900.16 | XP_026752518.2 uncharacterized protein LOC113512798 [*Galleria mellonella*] | 558 | -199356 |
| scaffold295.len631900.15 | RVE42842.1 hypothetical protein evm_012517 [*Chilo suppressalis*] | 237 | -221762 |
| scaffold295.len631900.14 | XP_026752518.2 uncharacterized protein LOC113512798 [*Galleria mellonella*] | 525 | -230909 |
| scaffold295.len631900.13 | XP_028032044.1 uncharacterized protein LOC114244433 [*Bombyx mandarina*] | 528 | -240183 |
| scaffold295.len631900.12 | XP_014359898.1 PREDICTED: uncharacterized protein LOC106711976 [*Papilio machaon*] | 582 | -299388 |
| scaffold295.len631900.11 | XP_026492948.1 uncharacterized protein LOC113398428 [*Vanessa tameamea*] | 564 | -318127 |
| scaffold295.len631900.10 | XP_028179081.1 tetra-peptide repeat homeobox protein 1-like [*Ostrinia furnacalis*] | 633 | -334774 |
| scaffold295.len631900.9 | XP_028179078.1 tetra-peptide repeat homeobox protein 1-like [*Ostrinia furnacalis*] | 687 | -365150 |
| scaffold295.len631900.8 | XP_026752831.1 serum response factor-binding protein 1-like [*Galleria mellonella*] | 396 | -402351 |
| scaffold295.len631900.7 | XP_023951243.1 membrane metallo-endopeptidase-like 1 [*Bicyclus anynana*] | 2478 | -413786 |
| scaffold295.len631900.6 | XP_028166528.1 cuticle secretory protein xP2-like [*Ostrinia furnacalis*] | 1044 | -433455 |
| scaffold295.len631900.5 | XP_026330726.1 delta-like protein 1 isoform X2 [*Hyposmocoma kahamanoa*] | 1026 | -446296 |
| scaffold295.len631900.4 | XP_026752691.1 alpha-(1,6)-fucosyltransferase [*Galleria mellonella*] | 1740 | -478838 |
| scaffold295.len631900.3 | XP_011566438.1 PREDICTED: protein maelstrom homolog [*Plutella xylostella*] | 1206 | -535112 |
| scaffold295.len631900.2 | XP_026741557.1 uncharacterized protein LOC113503694 [*Trichoplusia ni*] | 675 | -570674 |
| scaffold295.len631900.1 | XP_022826670.1 signal recognition particle receptor subunit alpha homolog [*Spodoptera litura*] | 438 | -592945 |
| scaffold298.len621769.8 | XP_028166538.1 zinc finger protein 2 homolog [*Ostrinia furnacalis*] | 1779 | -736762 |
| scaffold298.len621769.9 | XP_014359878.1 PREDICTED: LOW QUALITY PROTEIN: zinc finger protein 271-like [*Papilio machaon*] | 882 | -774050 |
| scaffold298.len621769.10 | XP_030034757.1 tubulin polyglutamylase TTLL6 [*Manduca sexta*] | 1986 | -827280 |
| scaffold298.len621769.11 | XP_026332591.1 GDP-mannose 4,6 dehydratase isoform X1 [*Hyposmocoma kahamanoa*] | 1086 | -851382 |
| scaffold298.len621769.12 | XP_026730896.1 uncharacterized protein LOC113496036 isoform X3 [*Trichoplusia ni*] | 1581 | -874971 |
| scaffold298.len621769.13 | XP_026493906.1 motile sperm domain-containing protein 1-like [*Vanessa tameamea*] | 642 | -885106 |
| scaffold298.len621769.14 | RVE42803.1 hypothetical protein evm_012560 [*Chilo suppressalis*] | 375 | -916005 |
| scaffold2118.len21571.1 | XP_034832589.1 probable tubulin polyglutamylase ttll-15 [*Aphantopus hyperantus*] | 780 | -941214 |
