# Supplementary material for: Genome-wide identification and functional analysis of long non-coding RNAs in Chilo suppressalis reveal their potential roles in chlorantraniliprole resistance
Source: Front Physiol. 2023 Jan 9;13:1091232. doi: 10.3389/fphys.2022.1091232 (PMC9868556; doi:10.3389/fphys.2022.1091232)
Supplement: Supplementary file 4 [file Table2.DOCX]

**Table S2.** Primers used for quantitative RT-PCR and RNAi.

| LincRNA ID | Forward primer (sense) (5’——3’) | Reverse primer (antisense) (5’——3’) | Product length (bp) |
| --- | --- | --- | --- |
| MSTRG.25316.8 | TGGGTTCTCAGAGTAATATTTACACC | AAGCAAGTATTTTAAACCTAACGGAT | 71 |
| MSTRG.7482.1 | ATCACGTCTTGGATATGGGC | GGGAACCTTAGCCGATTTCAG | 133 |
| MSTRG.25315.3 | GACAACTAGGTGCCTCAAGG | ACATGTCTGGCTTTATCCGG | 114 |
| MSTRG.3932.15 | CTGGCTTGGGTTAATGGTTTG | AAGGTGAAACGGTCTATGGC | 105 |
| MSTRG.3932.19 | CTGGCTTGTTAATGGTTTG | AAGGTGAAACGGTCTATGGC | 146 |
| MSTRG.17788.5 | GCTTAGACCGACATCGAACG | GAGACAGCTTCATTCCTCGG | 153 |
| MSTRG.22483.6 | GATTGTTTGATGTCGCGTCG | AGATCCACAGAACACAGAAAGATG | 127 |
| MSTRG.29805.1 | TGTTCACTGTTCAAGGTCAATTTT | TGGTGGAAACATAGATGCTTAAATC | 100 |
| Negative control | UUCUCCGAACGUGUCACGUTT | ACGUGACACGUUCGGAGAATT | siRNA |
| MSTRG.7482.1-231 | GCAGUGCUAUUAGUAUCUGTT | CAGAUACUAAUAGCACUGCTT | siRNA |
| Actin A1 (housing-keeping gene) | GTCGCTTCCCAAATTACATC | CTCCATATCGTTCCAGTCG | 178 |
