# Supplementary material for: Genome-wide identification and functional analysis of long non-coding RNAs in Chilo suppressalis reveal their potential roles in chlorantraniliprole resistance
Source: Front Physiol. 2023 Jan 9;13:1091232. doi: 10.3389/fphys.2022.1091232 (PMC9868556; doi:10.3389/fphys.2022.1091232)
Supplement: Supplementary file 5 [file Table5.DOCX]

**Table S5.** Esterase genes located on different chromosomes and dysregulated lncRNAs which were adjacent within 1000 kb of SSB.

| **Chromosome** | **Esterase genes** | **LncRNA genes dysregulated in R strain** | |
| --- | --- | --- | --- |
|  |  | **Up-regulated** | **Down-regulated** |
| Chr01 | chilo suppressalis isolate carboxylesterase CsuEst14; 25; 27; carboxyl/choline esterase CsuEst13 | —— | —— |
| Chr02 | chilo suppressalis isolate CsuEst10 carboxyl/choline esterase; esterase est2 | —— | —— |
| Chr04 | chilo suppressalis isolate carboxyl/choline esterase CsuEst12; 42; carboxylesterase CsuEst26 | MSTRG.21027.1, MSTRG.21076.40, MSTRG.21078.16 | MSTRG.21020.6, MSTRG.21076.13, MSTRG.21076.48 |
| Chr05 | antennal carboxylesterase 9; 18; chilo suppressalis isolate CsuEst3; 4; 23; 39; 49 | MSTRG.21506.1 | MSTRG.21503.1 |
| Chr07 | antennal carboxylesterase 4; chilo suppressalis isolate CsuEst16 | MSTRG.23779.1 | —— |
| Chr08 | antennal carboxylesterase 7; 14; chilo suppressalis isolate CsuEst1 antennal esterase; CsuEst18 carboxylesterase | —— | —— |
| Chr09 | chilo suppressalis isolate CsuEst35 antennal esterase | MSTRG.25727.1 | —— |
| Chr11 | esterase est4; antennal carboxylesterase 16; chilo suppressalis isolate CsuEst2 antennal esterase; CsuEst22 carboxyl/choline esterase | MSTRG.3040.1, MSTRG.3044.1 | MSTRG.3035.1, MSTRG.3056.1, MSTRG.3155.2 |
| Chr12 | antennal carboxylesterase 3; chilo suppressalis isolate CsuEst43 antennal esterase | —— | MSTRG.4188.1 |
| Chr13 | chilo suppressalis isolate CsuEst20 carboxylesterase | —— | —— |
| Chr15 | antennal carboxylesterase 15; chilo suppressalis isolate CsuEst32 carboxylesterase; CsuEst37 carboxyl/choline esterase; juvenile hormone esterase CsuEst7; 40; CsuEst46 juvenile hormone esterase precursor | —— | MSTRG.6546.7; MSTRG.6577.1; MSTRG.6578.1; MSTRG.6647.1 |
| Chr17 | antennal carboxylesterase 2; 13; chilo suppressalis isolate CsuEst28 antennal esterase; carboxylesterase CsuEst17; 21; 38 | MSTRG.8735.1 | MSTRG.8748.1 |
| Chr19 | esterase est3; antennal carboxylesterase 8; 17; chilo suppressalis isolate antennal esterase CsuEst6; 19; 24; carboxylesterase CsuEst50; 51; carboxyl/choline esterase CsuEst9; 34 | —— | —— |
| Chr22 | alphe-esterase EST36; antennal esterase; antennal carboxylesterase 11; 12; chilo suppressalis isolate antennal esterase CsuEst36; 45 | —— | MSTRG.13793.2, MSTRG.13837.2 |
| Chr23 | antennal carboxylesterase 19; 20; chilo suppressalis isolate antennal esterase CsuEst5; 8; CsuEst30 carboxylesterase | MSTRG.14567.2 | —— |
| Chr29 | antennal carboxylesterase 5; 6; chilo suppressalis isolate carboxyl/choline esterase CsuEst11; 15 | MSTRG.18348.1, MSTRG.18366.2 | —— |
