# Supplementary material for: Genome-wide identification and functional analysis of long non-coding RNAs in Chilo suppressalis reveal their potential roles in chlorantraniliprole resistance
Source: Front Physiol. 2023 Jan 9;13:1091232. doi: 10.3389/fphys.2022.1091232 (PMC9868556; doi:10.3389/fphys.2022.1091232)
Supplement: Supplementary file 6 [file Table3.DOCX]

**Table S3.** Library sequencing and assembly of 6 RNA-seq samples from susceptible and resistant strains of SSB against chlorantraniliprole.

| Library ID | Clean Base (G) | Clean Reads | Rate of alignment to genome (%) | Assembled transcripts number | Merged transcripts |
| --- | --- | --- | --- | --- | --- |
| S1 | 17.37 | 51,627,732 | 91.85 | 93,442 | 40,573 |
| S2 | 14.79 | 43,490,487 | 90.60 | 99,505 |  |
| S3 | 15.08 | 43,801,451 | 90.47 | 88,241 |  |
| R1 | 15.16 | 50,532,425 | 89.13 | 163,143 |  |
| R2 | 16.54 | 55,131,982 | 88.46 | 147,488 |  |
| R3 | 15.79 | 52,635,860 | 86.00 | 140,169 |  |
