# Supplementary material for: Genome-wide identification and functional analysis of long non-coding RNAs in Chilo suppressalis reveal their potential roles in chlorantraniliprole resistance
Source: Front Physiol. 2023 Jan 9;13:1091232. doi: 10.3389/fphys.2022.1091232 (PMC9868556; doi:10.3389/fphys.2022.1091232)
Supplement: Supplementary file 7 [file Table4.DOCX]

**Table S4.** P450 genes located on different chromosomes and dysregulated lncRNAs which were adjacent within 1000 kb of SSB.

| **Chromosome** | **CYP genes** | **LncRNA genes dysregulated in R strain** | |
| --- | --- | --- | --- |
|  |  | **Up-regulated** | **Down-regulated** |
| Chr01 | CYP4G90, CYP4G91, CYP4G92, CYP301B1, CYP333B26, CYP6AB50, CYP6AB52 | MSTRG.349.1, MSTRG.415.1 | MSTRG.1412.5 |
| Chr02 | CYP303A1, CYP305-1A1, CYP305B1, CYP340-79, CYP340AK1 | MSTRG.11680.1 | —— |
| Chr04 | CYP4M38, CYP4M39, CYP4M84 | —— | —— |
| Chr05 | CYP321F1, CYP321F2, CYP321F3, CYP321F4 | —— | —— |
| Chr06 | CYP306A1, CYP6AW1, CYP18A1 | MSTRG.22984.1 | MSTRG.22889.1 |
| Chr08 | CYP302A1, CYP341-51, CYP341-91, CYP341A15, CYP341A57, CYP341B10, CYP341B34 | MSTRG.24622.1 | MSTRG.24919.2 |
| Chr09 | CYP304F13, CYP4L27 | MSTRG.25862.1 | —— |
| Chr10 | CYP9A11, CYP9A12, CYP9A42, CYP9A59, CYP9A68, CYP9A69, CYP9A77 | MSTRG.2603.2 | MSTRG.2756.1, MSTRG.2758.1, MSTRG.2776.2 |
| Chr11 | CYP315A1, CYP333A9, CYP354A12, CYP428A1 | MSTRG.3379.1 | MSTRG.3390.5, MSTRG.3290.3 |
| Chr12 | CYP15C1 | MSTRG.4303.1, MSTRG.4360.1 | —— |
| Chr14 | CYP324A12, CYP6AB45, CYP6AB47, CYP6AB48 | MSTRG.5759.3, MSTRG.6379.1 | —— |
| Chr15 | CYP6CT1 | —— | —— |
| Chr16 | CYP339A1, CYP6AB49 | —— | —— |
| Chr17 | CYP6CV4, CYP6CV5, CYP6AB46 | MSTRG.8331.1 | MSTRG.8334.3 |
| Chr18 | CYP4AU11, CYP4AU10 | —— | —— |
| Chr20 | CYP367-96, CYP367A9, CYP367B9, CYP367B, CYP6AE60, CYP6AE61 | MSTRG.12713.1 | MSTRG.12637.1, MSTRG.12638.1, MSTRG.12639.1, MSTRG.12641.1, MSTRG.12699.1, MSTRG.12717.1 |
| Chr21 | CYP314A1 | MSTRG.13106.2 | MSTRG.13132.1 |
| Chr23 | CYP6AB51 | MSTRG.14513.1, MSTRG.14532.1, MSTRG.14544.1, MSTRG.14543.2 | —— |
| Chr26 | CYP6-1, CYP6-2 | MSTRG.16321.4 | MSTRG.16292.1 |
| Chr27 | CYP307A2 | —— | MSTRG.17206.2 |
