# Supplementary material for: Genome-wide identification and functional analysis of long non-coding RNAs in Chilo suppressalis reveal their potential roles in chlorantraniliprole resistance
Source: Front Physiol. 2023 Jan 9;13:1091232. doi: 10.3389/fphys.2022.1091232 (PMC9868556; doi:10.3389/fphys.2022.1091232)
Supplement: Supplementary file 8 [file Table6.DOCX]

**Table S6.** ATP-binding cassette transporter (ABC) genes located on different chromosomes and dysregulated lncRNAs which were adjacent within 1000 kb of SSB.

| **Chromosome** | **ABC genes** | **LncRNA genes dysregulated in R strain** | |
| --- | --- | --- | --- |
|  |  | **Up-regulated** | **Down-regulated** |
| Chr01 | A4 | —— | —— |
| Chr02 | B2, F2, H2, H4 | MSTRG.11059.1, MSTRG.11059.2, MSTRG.11060.1, MSTRG.11060.2, MSTRG.11060.3 | MSTRG.10737.1, MSTRG.11388.1 |
| Chr04 | G13, G14 | —— | MSTRG.20926.2 |
| Chr05 | C2, C3, C4, C7, C8, C10, F1 | MSTRG.21399.1, MSTRG.21734.2 | MSTRG.21723.3 |
| Chr06 | C6, F3, G15 | —— | —— |
| Chr07 | C1, C9, G1, G3, G4, G5, G6, G7, G8, G9, G10 | MSTRG.23779.1 | MSTRG.23909.1, MSTRG.24212.1 |
| Chr08 | B4, D3, G12 | MSTRG.25315.1, MSTRG.25315.2, MSTRG.25315.3, MSTRG.25316.1, MSTRG.25315.5, MSTRG.25315.6, MSTRG.25315.7, MSTRG.25315.8, MSTRG.25316.2, MSTRG.25316.4, MSTRG.25315.10, MSTRG.25316.8, MSTRG.25316.9, MSTRG.25309.1, MSTRG.25309.2, MSTRG.25309.3, MSTRG.25310.3, MSTRG.25310.5, MSTRG.25310.6 | MSTRG.25346.2 |
| Chr10 | A2, A3, H1, H3 | MSTRG.2544.2 | MSTRG.2123.1, MSTRG.2544.10 |
| Chr11 | B3 | MSTRG.3256.1 | MSTRG.3290.3 |
| Chr12 | G2 | —— | MSTRG.4188.1 |
| Chr16 | B6 | MSTRG.7506.2 | MSTRG.7518.1 |
| Chr19 | B7 | —— | —— |
| Chr20 | A5 | MSTRG.12252.2, MSTRG.12253.1 | —— |
| Chr21 | E1 | —— | MSTRG.13132.1 |
| Chr24 | D2 | —— | MSTRG.15193.3, MSTRG.15223.1, MSTRG.15224.1 |
| Chr29 | C5 | MSTRG.18348.1, MSTRG.18366.2, MSTRG.18369.2 | —— |
